# Transcriptional regulation of IFN-λ genes in Hepatitis C virus-infected hepatocytes via IRF-3·IRF-7·NF-κB complex

**DOI:** 10.1186/2051-1426-2-S3-P173

**Published:** 2014-11-06

**Authors:** Hai-Chon Lee, Je-In Youn, Kyungwha Lee, Hwanyul Yong, Seung-Yong Seong

**Affiliations:** 1 Wide River Institute of Immunology, Seoul National University, Republic of Korea

## 

**Figure 1:**
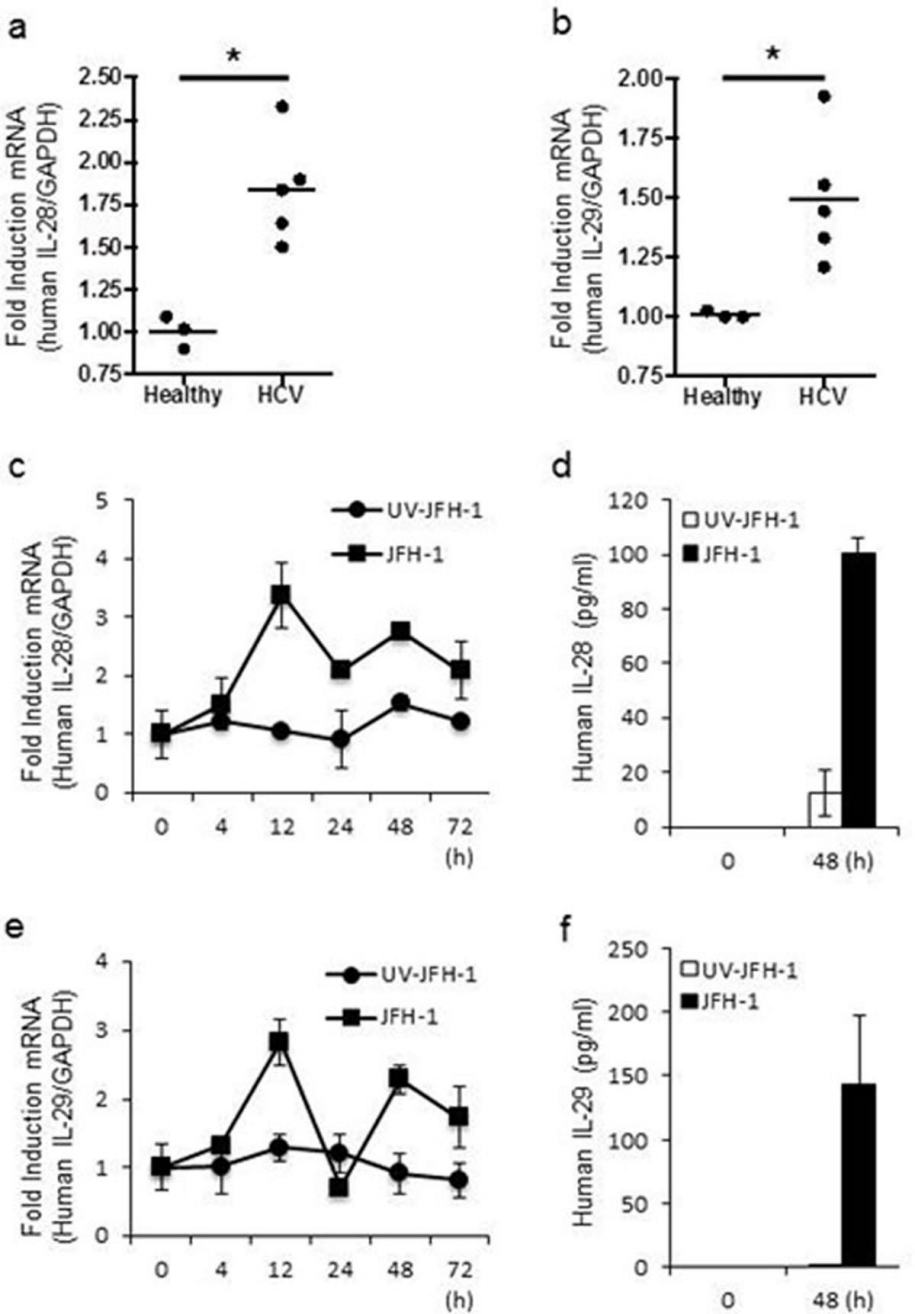
**Hepatic induction of IFN-λ genes during HCV infection**. A, B. Total RNA was extracted from liver tissues of healthy and chronic HCV patients for IL-28A/B (A) and IL-29 (B) mRNA quantification by real-time PCR. C-F. Human primary hepatocytes were inoculated with UV-irradiated JFH-1 or JFH-1 for the indicated amounts of time, and IFN-λ gene levels were quantified by real-time PCR (C, E) while protein levels were analyzed by ELISA (D, F). Data represent means ± SD of three independent experiments (p<0.01).

**Figure 2:**
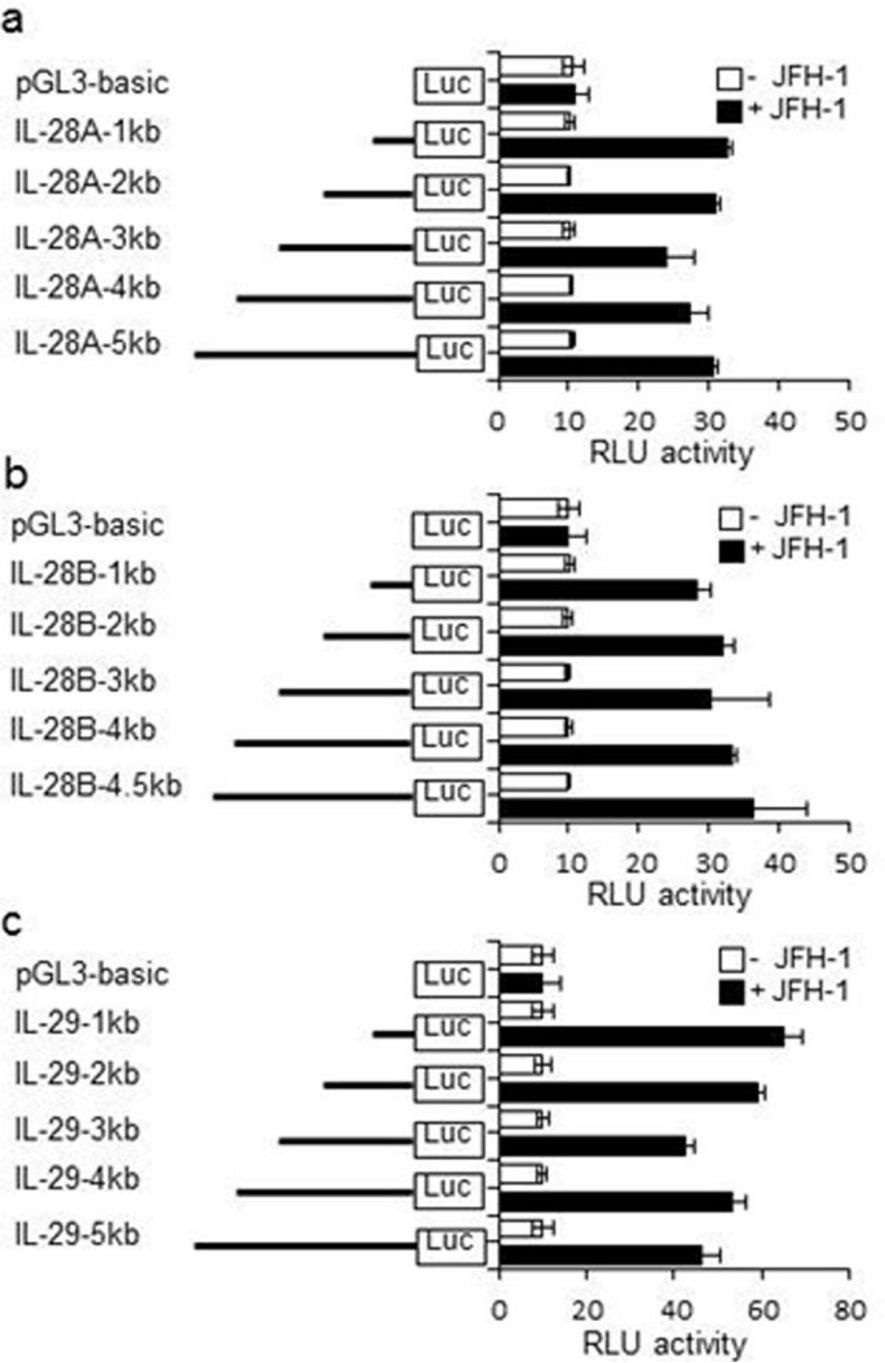
**Transcriptional activity at the IL-28A, IL-28B, and IL-29 promoter by HCV infection**. PH5CH8 cells were transfected with a mixture of the luciferase constructs containing various 5' deletions of the human IL-28A (A), IL-28B (B), or IL-29 (C) promoters and Renilla luciferase control vector. The cells were cultured for 18 hours and then infected with JFH-1 for 12 hours. Luciferase activity in whole cell lysates was normalized to Renilla luciferase activity. Data are the mean ± SD of triplicate data points from five independent experiments.

**Figure 3:**
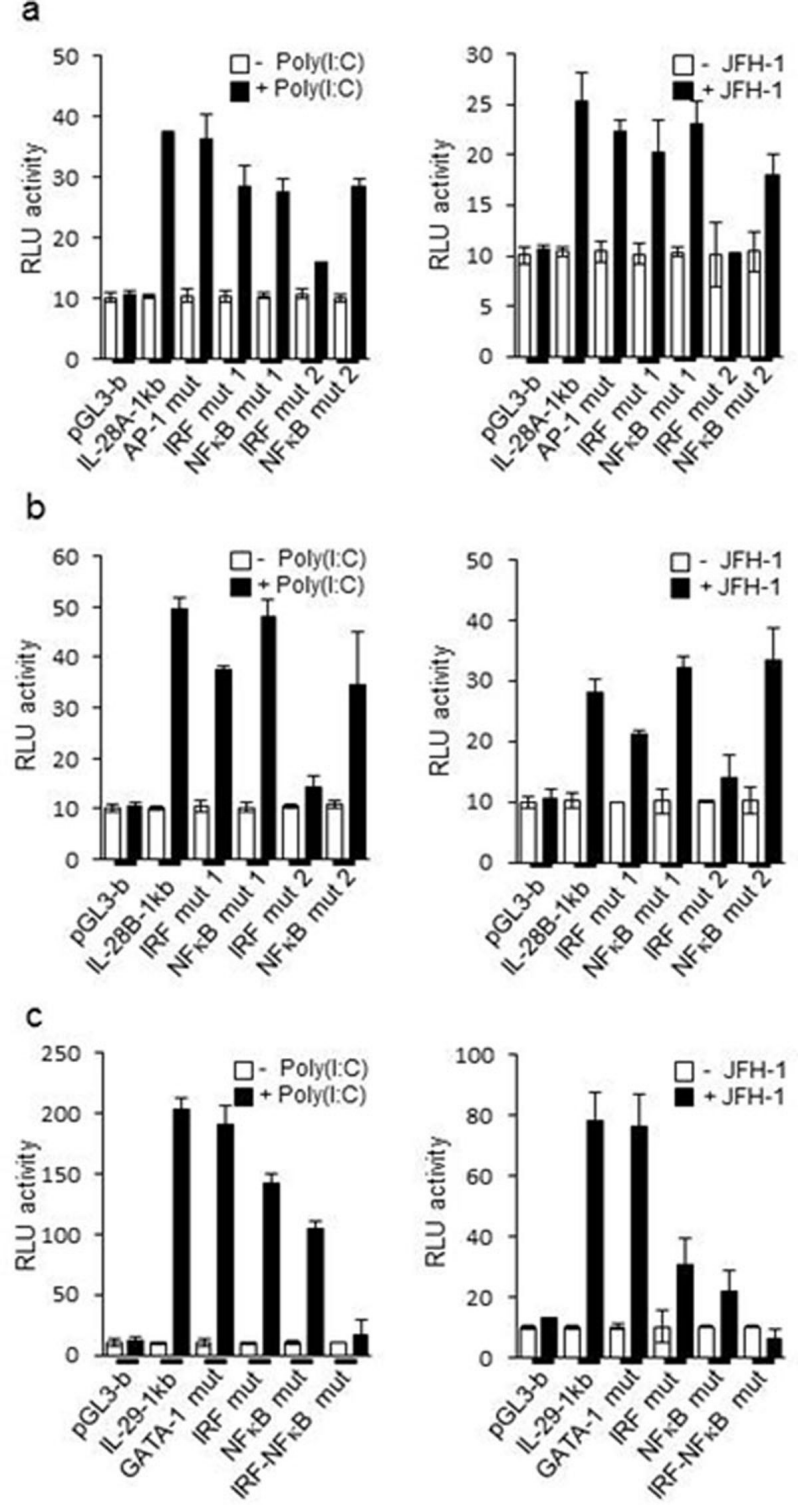
**IRF-7, and NF-κB differentially determine expression of IL-28A/B and IL-29**. Nucleotide sequence from 1.0 kb upstream of the transcription start site of human IL-28A, IL-28B, and IL-29. The positions of the IRF, NF-κB, AP-1, and GATA-1 binding sites are indicated. (B-D) PH5CH8 cells were transiently transfected with control plasmid pGL3-basic, and plasmids encoding luciferase constructs containing human IL-28A (pGL3-IL-28A 1kb) (B), IL-28B (pGL3-IL-28B 1kb) (C), or IL-29 (pGL3-IL-29 1kb) (D) promoter regions. Various mutation constructs of the above promoter regions were also transfected into PH5CH8 cells, using a Renilla luciferase vector as a control. The cells were cultured for 24 hours and then stimulated with poly(I:C) for 6 h or infected with JFH-1 for 12 h. Luciferase activity in whole cell lysates was normalized to Renilla luciferase activity. Data are the mean ± SD of triplicate data points from five independent experiments.

**Figure 4:**
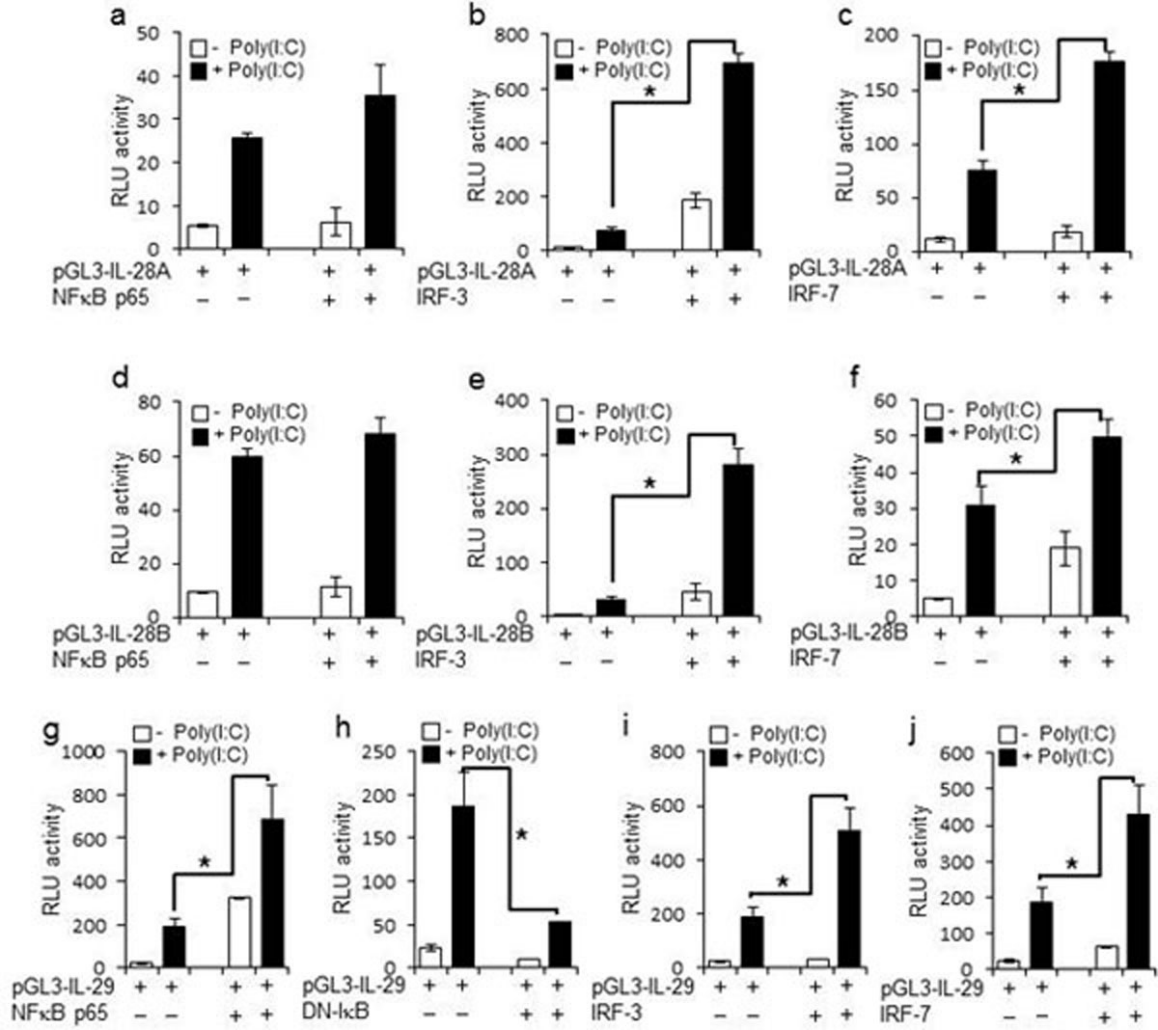
**Overexpression of IRFs and NF-κB confirms their role in expression of IFN-λ genes**. PH5CH8 cells were transiently transfected with plasmids encoding different promoter regions of human IL-28A (pGL3-IL-28A 1kb) (A-C), IL-28B (pGL3-IL-28B 1kb) (D-F), or IL-29 (pGL3-IL-29 1kb) (G-J), and expression vectors encoding IRF-3, IRF-7, and NF-κB or a dominant-negative mutant of IKKβ (DNIκB). At 18 h post-transfection, cells were stimulated with poly(I:C) for 6 hours. Luciferase activity in whole cell lysates was normalized to Renilla luciferase activity. Data represent mean ± SD of triplicate data points of one experiment and are representative of five independent experiments (p<0.01).

**Figure 5:**
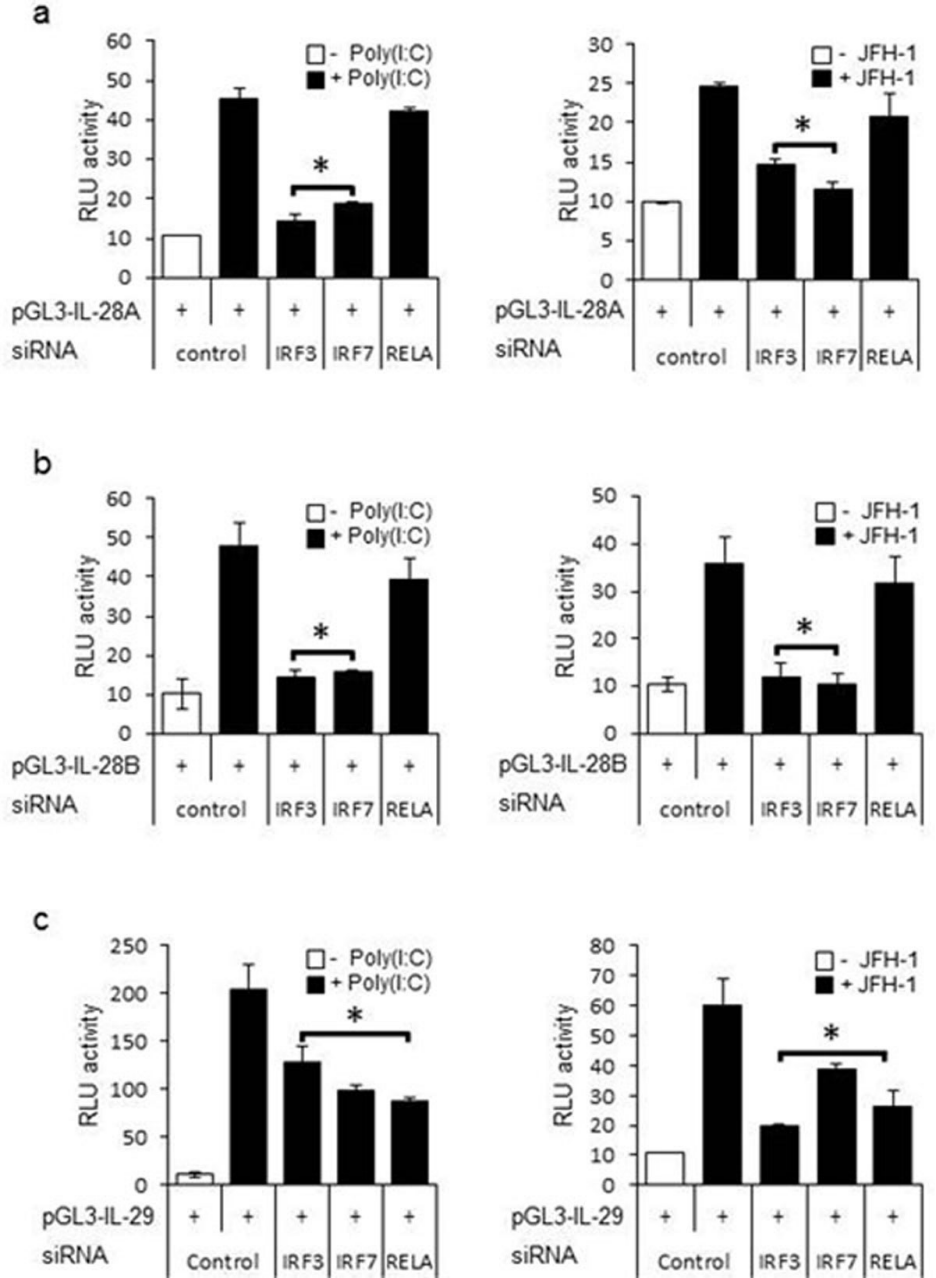
**IRFs are core transcription factors of IFN-λ genes**. IL-28B (B), or IL-29 (C) in combination with siRNA specific for IRF-3, IRF-7, RELA(NF-κB p65), or control. At 36 h post-transfection, cells were stimulated with poly(I:C) for 6 hours or infected with JFH-1 for 12 h. Luciferase activity in whole cell lysates was normalized to Renilla luciferase activity. Data represent mean ± SD of triplicate data points of one experiment and are representative of three independent experiments. Statistical significance (p<0.01) is relative to control + poly(I:C) or control + JFH-1.

**Figure 6:**
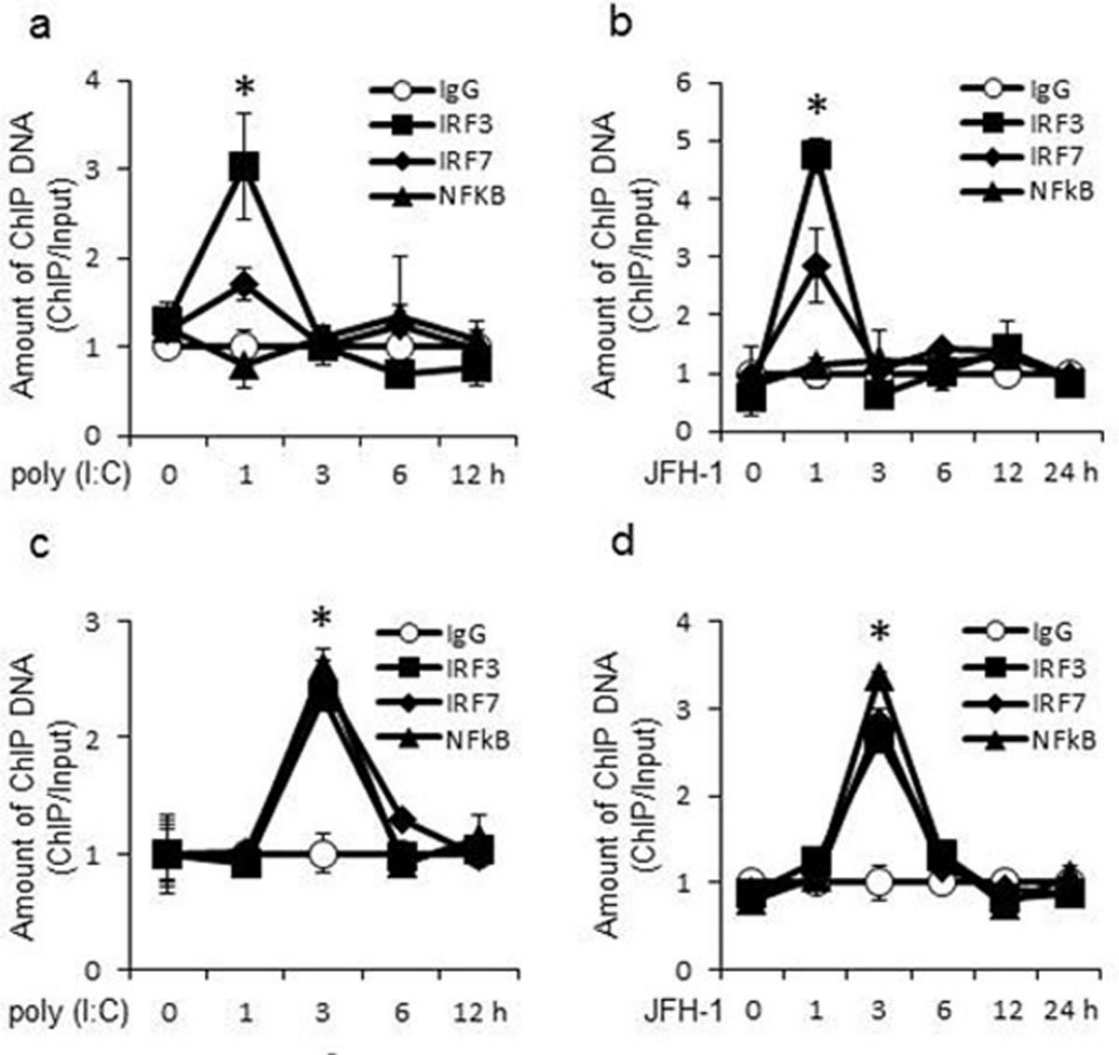
**IRFs and NF-κB directly bind the promoters of IFN-λ genes**. PH5CH8 cells were stimulated with poly(I:C) (A and C) or infected with JFH-1 (B and D) for the indicated lengths of time. Chromatin was immunoprecipitated with anti-IRF-3, anti-IRF-7, anti-NF-κB antibody or control normal rabbit IgG. Purified ChIP and input DNA were analyzed by real-time quantitative PCR. The amount of ChIP DNA was normalized to that of input DNA. The mean value of control antibody before stimulation was arbitrarily defined as 1. Data are the mean ± SD of triplicate data points from a representative of five independent experiments. Statistical significance (p<0.01) is relative to IgG.

**Figure 7:**
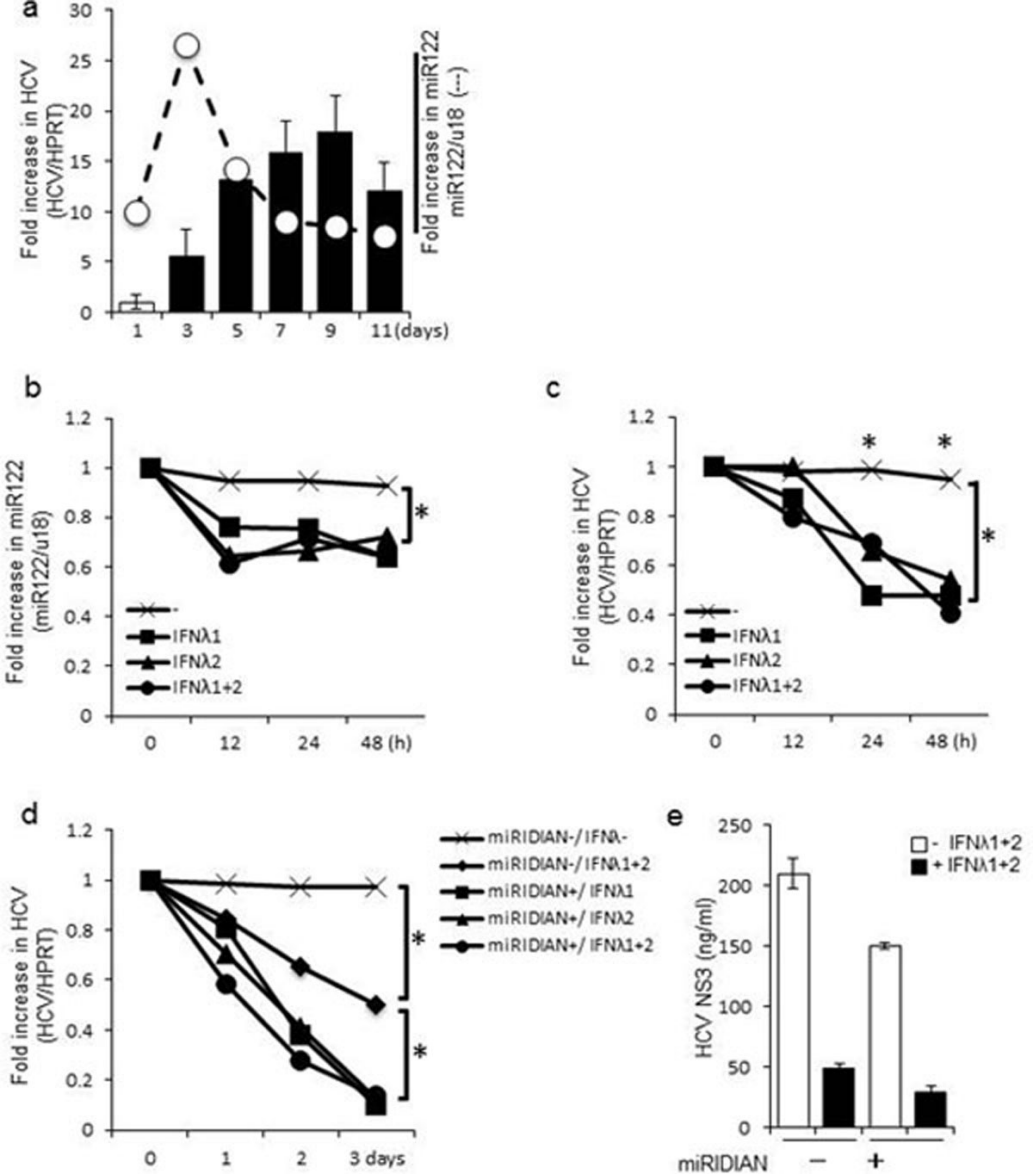
**miR-122-dependent replication of HCV is suppressed by IFN-λ stimulation**. Huh 7.5.1 cells infected with JFH-1 for the indicated number of days and HCV RNA or miR-122 levels were quantified by real time PCR. The mean value of day 1 post-infection was arbitrarily defined as 1 in fold increase in HCV, or as 10 in fold increase in miR-122 (A). Cells were infected with JFH-1 for 5 days, and then replated in 6-well plates. After 24 hours, the HCV-infected cells were stimulated with recombinant IFN-λ (100 ng) for 0-48 hours. The amount of HCV RNA or miR-122 was quantified by real time PCR (B, C). Similarly, HCV-infected cells were replated in 6-well plates after 5 days of infection. After 24 hours, cells were transfected with miRIDIAN or negative control miRIDIAN (20 pmol) for 36 h, and stimulated with or without IFN-λ (100 ng each) for 1-3 days. HCV RNA was quantified by real time PCR (D) while HCV NS3 protein was measured by ELISA (E). Data shown are from one experiment and are representative of three experiments with similar results. Statistical significance (p<0.01) is relative to the control group that received no treatment.

**Figure 8:**
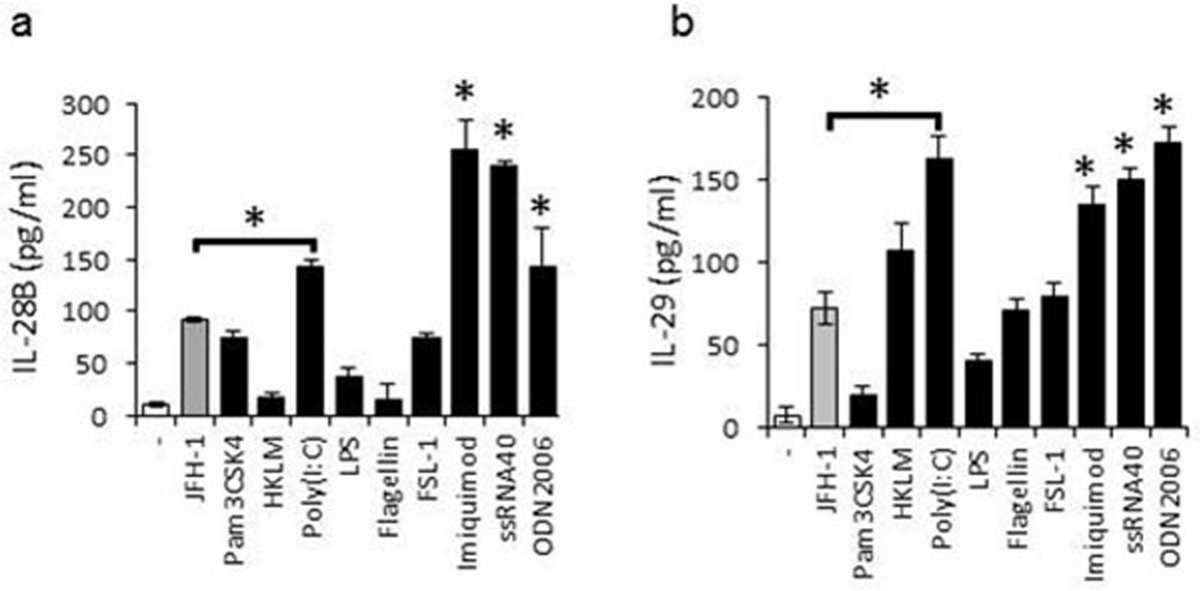
**TLR3, 7, 8, and 9 induce human IFN-λ gene expression in human primary hepatocytes**. Human primary hepatocytes were incubated with JFH-1, Pam3CSK4 (1 μg/ml), HKLM (108 cells/ml), Poly (I-C) (5 μg/ml), LPS (10 μg/ml), Flagellin (1 μg/ml), FSL-1 (100 ng/ml), Imiquimod (1 μg/ml), ssRNA (1 μg/ml), or ODN2006 (5μM) for 24 hours. (A) Toll-like receptors (TLR) and their respective ligands. Supernatants were harvested and IL-28B (B) and IL-29 (C) were measured by ELISA. Data are the mean ± SD of triplicate data points from one experiment. Results are representative of three independent experiments. Statistical significance (p<0.01) is relative to JFH-1 infection.

Hepatitis C virus (HCV) infection in hepatocytes stimulates innate antiviral responses including the production of type III interferons (IFN-λ), including IL-28A, IL-28B, and IL-29 (Figure 1). However, the molecular mechanism(s) regulating the expression of *IFN-*λ genes in HCV-infected hepatocytes remains undefined. In this study, we examined regulatory elements involved in the induction of *IFN-*λ genes following HCV infection in hepatocytes and further determined the binding of specific transcription factor(s) to promoter regions of *IFN-*λ genes. Our studies reveal that the regulatory portion for *IL-28A, IL-28B*, and*IL-29 *genes is localized to a 1-kb region in their respective promoters (Figure 2, 4). Notably, interferon regulatory factor (IRF)-3 and -7 are the key transcriptional factors for the induction of *IL-28A *and *IL-28B*genes (Figure 5, 6), whereas NF-κB is an additional requirement for the induction of the *IL-29 *gene (Figure 3). Ligation of Toll-like receptors (TLR) 3, 7, 8, and 9, which also activate IRFs and NF-κB, resulted in more robust production of IFN-λ than that observed with HCV infection, verifying the importance of TLR pathways in IFN-λ production (Figure 8). Furthermore, the addition of IFN-λ to HCV-infected hepatocytes decreased viral replication and produced a concurrent reduction in microRNA-122 (miR-122). The decrease in viral replication was enhanced by the co-administration of IFN-λ and miR-122 inhibitor (miRIDIAN) (Figure 7), suggesting that such combinatorial therapies may be beneficial for the treatment of chronic HCV infection.

